# Localized primary renal aspergillosis in a diabetic patient following lithotripsy – a case report

**DOI:** 10.1186/1471-2334-7-58

**Published:** 2007-06-14

**Authors:** Jalaluddin A Haq, Mohammad AM Khan, Nazma Afroze, Tahniyah Haq

**Affiliations:** 1 Department of Microbiology, Bangladesh Institute of Research and Rehabilitation in Diabetes, Endocrine and Metabolic Disorders, 122, Kazi Nazrul Islam Avenue, Dhaka, Bangladesh; 2 School of Health Sciences, University Sains Malaysia, Kubang Kerian 16150, Kelantan, Malaysia; 3 Department of Urology, Bangladesh Institute of Research and Rehabilitation in Diabetes, Endocrine and Metabolic Disorders, 122, Kazi Nazrul Islam Avenue, Dhaka, Bangladesh; 4 Department of Pathology, Bangladesh Institute of Research and Rehabilitation in Diabetes, Endocrine and Metabolic Disorders, 122, Kazi Nazrul Islam Avenue, Dhaka, Bangladesh; 5 DCCS unit, Bangladesh Institute of Research and Rehabilitation in Diabetes, Endocrine and Metabolic Disorders, 122, Kazi Nazrul Islam Avenue, Dhaka, Bangladesh

## Abstract

**Background:**

Primary renal aspergillosis is rare in diabetic patients. Diagnosis of localized primary renal *Aspergillus *infection in diabetic patients requires careful investigations due to its benign presentation and lack of associated systemic clinical features. There is also paucity of information on the role of conservative treatment of such localized infection with antifungal agents only. Here, we describe a case of localized renal aspergillosis in a type 2 diabetic patient with a brief review of literature.

**Case presentation:**

We describe a case of unilateral renal aspergillosis following intracorporeal pneumatic lithotripsy (ICPL) in a type 2 diabetic man. The patient presented with mild pain in the left lumbar region and periodic expulsion of whitish soft masses per urethra, which yielded growth of *Aspergillus fumigatus*. He was treated initially with amphotericin B; however, it was stopped after 2 weeks, as he could not tolerate the drug. Subsequently, he was successfully treated with oral itraconazole.

**Conclusion:**

Localized renal aspergillosis may be suspected in diabetic patients having history of urinary tract instrumentation, mild lumbar pain, passage of suspicious masses in urine and persistent pyuria. Examination of the suspicious substances expelled per urethra is essential for diagnosis as routine multiple urine analysis may yield negative results. Conservative treatment with oral itraconazole alone is effective in cases with incomplete obstruction.

## Background

Renal aspergillosis is a rare entity. Patients with compromised immune status, such as diabetics, those on corticosteroid therapy and HIV positive individuals are more vulnerable to infection by *Aspergillus *species [1]. A few cases of renal aspergillosis have been reported in diabetics [2-6]. Here, we describe a case of localized renal aspergillosis following ICPL and ureteric stenting in a diabetic man. He was successfully treated with oral itraconazole without surgical intervention.

## Case presentation

A 45-year-old man with type 2 diabetes was hospitalized with complaints of mild pain in the left lumbar region, irregular low-grade fever and occasional dysuria for 3 months. He occasionally passed whitish soft masses along with urine for last two months and his loin pain aggravated prior to passage of such soft masses per urethra. Three months prior to hospitalization, he underwent ureteroscopy and ICPL for removal of stone in left kidney. A stent was then placed in left ureter following ICPL. However, the stent was removed after 2 weeks as he developed fever and lower abdominal pain, which did not respond to antibiotic treatment. On physical examination, his vital signs were normal. There was no lymphadenopathy, organomegaly or renal angle tenderness. Repeated routine urine analysis revealed persistent pus cells (40–50 cells/hpf) and occasional erythrocytes. Urine cultures were negative for bacteria and fungi on three consecutive samples. No acid-fast bacilli were detected by Z-N stain in three consecutive morning urine samples. Ultrasound scan and intravenous urogram (IVU) revealed a cystic lesion (4 × 3.9 cm) at lower pole cortical region of the left kidney. Chest X-ray and tuberculin test were negative. Blood culture was negative and count was within normal limits (Hb14.6 g/dl, total leukocyte count 10.8 × 10^9^/L, neutrophils 57%, lymphocytes 33%, monocytes 04% and eosinophils 10%, erythrocyte sedimentation rate 25 mm 1^st ^hour, total platelet count 288 × 10^9^/L). His serum creatinine, urea, electrolytes and liver function tests were all within normal range. Initially, his fasting blood glucose level was 10 mmol/L and he was on oral hypoglycaemic agent. His diabetes was controlled with insulin and fasting blood sugar was maintained between 5.5–6.5 mmol/L throughout the treatment period. A course of antibiotic (ciprofloxacin 500 mg twice daily for 7 days) was given without resolution of the symptoms.

**Figure 1:**
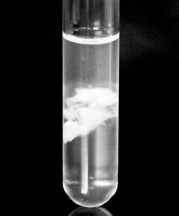
Soft whitish mass (Fungal mass) passed in urine.

Subsequently, microscopic examination of the soft whitish masses (Fig. 1), which was expelled along with urine revealed a mesh of fungal hyphae and upon culture yielded growth of *Aspergillus fumigatus*. The case was diagnosed as a localized aspergillosis of the urinary tract.

Monotherapy with amphotericin B was planned and accordingly the treatment was initiated with intravenous amphotericin B 1.25 mg/kg/day. However, amphotericin B had to be stopped after 2 weeks of treatment due to rise in creatinine level, vomiting and weakness. Oral itraconazole (200 mg twice daily) was started. After one month, he became symptom free and he ceased to pass whitish soft masses (fungal ball) per urethra.

Itraconazole 200 mg twice daily was continued for a total of two months and thereafter, the dose was reduced to 100 mg twice daily and continued for another four months. He was followed regularly for 18 months. Repeat IVU and ultrasound scan revealed resolution of cystic lesion. He was symptom free 17 months after initiation of itraconazole, and 12 months after stoppage of itraconazole. His renal function, liver function, routine urine analysis and culture were all normal. No surgical intervention was required.

## Discussion

The rate of urinary tract infection following ICPL and ureteric stenting is between 1 to 2% and around 3.5% respectively and is mainly due to gram-negative bacteria [7-9]. So far, there is no report of *Aspergillus *infection of urinary tract following ICPL. Aspergillosis of urinary tract may occur by three ways namely, by ascending infection from the lower tract, from haematogenous dissemination or due to *Aspergillus *cast in renal pelvis [10]. Renal aspergillosis due to haematogenous dissemination is the most common while localized infection is rare [10,11]. In our case, no history of urinary tract infection and passage of fungal masses per urethra prior to ICPL and instrumentation suggest that the *Aspergillus *was introduced into the urinary tract during ureteroscopy and placement of stent in the ureter. Probably, fungal spores contaminated the instrument during the procedure or the sterilization of the equipment was inadequate. The infection was a primary and localized aspergillosis of the urinary tract as no other known focus of fungal infection was present elsewhere in the body. Probably, the initial focus of infection originated in renal cortical tissue, which later extended and opened into the pelvis or vice versa. The cystic lesion observed in lower pole cortical region of the left kidney and passage of fungal balls in urine indicated that it could be a renal pelvis type aspergillosis as described by Denning [12]. Renal stone, local tissue damage due to ICPL and underlying diabetes facilitated the localized infection. Initial multiple routine urine analysis and culture failed to detect fungal hyphae and isolate *Aspergillus *until the whitish soft masses expelled per urethra were examined. In our patient, infection resolved completely with oral itraconazole and did not relapse after 18 months though poor response to itraconazole in renal aspergillosis in diabetic patient was noted [13].

Recently, improved outcome in invasive aspergillosis has been reported by sequential therapy with amphotericin B and itraconazole [14]. In our case, we planned monotherapy with amphotericin B and itraconazole was only given when the patient did not tolerate amphotericin B. Moreover, the patient continued to pass fungal balls during amphotericin B and in first month of itraconazole therapy. Therefore, we believe that initial amphotericin B for two weeks was unable to clear the infection. Patient only became symptom free and culture negative one month after itraconazole therapy. Therefore, we assume that though our patient received amphotericin B and itraconazole sequentially, in fact, the response was due to itraconazole. However, we cannot entirely rule out the beneficial effects of prior amphotericin B therapy as it has been shown that the use of amphotericin B and itraconazole in sequence has synergistic effects and improved outcome [14]. Moreover, there is a lack of specific standardized sequential regime of amphotericin B and itraconazole for each type of invasive aspergillosis except for neutropenic patients [15]. Most important problem being the optimum duration of initial amphotericin B treatment before itraconazole can be started. Therefore, the two weeks amphotericin B therapy that our patient has received may or may not be adequate to have an optimum effect on subsequent outcome.

Analysis of a large series of patients revealed that *Aspergillus *infection of the kidney constitutes about 30% (27/90) of total renal fungal infection and majority (63%) results from disseminated infection [10]. Renal *Aspergillus *infection usually results from disseminated fungal infection having underlying immunocompromised states. Co-existing conditions include malignancy, use of chemotherapeutic and immunosuppressive agents, transplantation, liver diseases, AIDS and diabetes [1,10,11]. However, primary renal aspergillosis in diabetic patients is rare. So far, 11 cases of renal *Aspergillus *infection in diabetic patients have been reported between 1962–2006 [1-6,13,16-19]. Majority (63%) was male and 72% (8/10) had unilateral involvement. *A. flavus *and *A. fumigatus *were equally and most frequently isolated species. Clinical features ranged from benign presentation to obstruction of urinary tract and systemic complications. No typical symptoms of urinary tract infection may be present. Fungal ball or bezoar was present in 63% cases. Multiple routine examinations of urine and cultures may be negative unless careful attention is given to the fungal masses expelled along with urine. Systemic antifungal drug (amphotericin B) with or without local irrigation and surgical intervention have been the mainstay of treatment. However, successful outcome of renal aspergillosis with conservative treatment alone has also been reported [4,20]. Treatment of diabetic patients having compromised renal function is difficult with amphotericin B. In such cases, use of oral itraconazole in adequate dose as reported earlier and shown in our case is a good alternative [19,21]. Use of oral itraconazole even reduces the duration of hospital stay and has better compliance due to less adverse effects. Voriconazole and echinocandins (caspofungin) are new antifungal agents with broad activity against wide variety of fungus including *Aspergillus *sp [22,23]. These agents have improved outcome and less toxicity compared to amphotericin B in invasive aspergillosis. However, these agents are expensive and the daily cost of therapy for voriconazole is US$ 67 compared to US$ 18–37 for itraconazole [24].

## Conclusion

Our case suggests that localized renal aspergillosis may be suspected when diabetic patients having history of urinary tract instrumentation present with passage of suspicious masses in urine. Specific examination of the suspicious substance passed per urethra is essential for diagnosis of renal aspergillosis as routine multiple urine analysis may yield negative results due to intermittent excretion of fungus in urine.

## Competing interests

The author(s) declare that they have no competing interests.

## Authors' contributions

JAH was involved in diagnosis, management and preparation of the manuscript. MAMK was involved in patient management and follow up. NA was involved in diagnosis of the case. TH reviewed the case notes, carried out the literature review and prepared the initial manuscript.

## Pre-publication history

The pre-publication history for this paper can be accessed here:


